# Consistent Changes in Cortico-Subthalamic Directed Connectivity Are Associated With the Induction of Parkinsonism in a Chronically Recorded Non-human Primate Model

**DOI:** 10.3389/fnins.2022.831055

**Published:** 2022-03-04

**Authors:** Joyce Chelangat Bore, Carmen Toth, Brett A. Campbell, Hanbin Cho, Francesco Pucci, Olivia Hogue, Andre G. Machado, Kenneth B. Baker

**Affiliations:** ^1^Department of Neurosciences, Lerner Research Institute, Cleveland Clinic, Cleveland, OH, United States; ^2^Department of Biomedical Engineering, Case Western Reserve University, Cleveland, OH, United States; ^3^Center for Neurological Restoration, Cleveland Clinic, Neurological Institute, Cleveland, OH, United States; ^4^Department of Neurosurgery, Cleveland Clinic, Neurological Institute, Cleveland, OH, United States

**Keywords:** Parkinson’s disease, connectivity, subthalamic nucleus, electrophysiology, Granger causality, coherence, beta band

## Abstract

Parkinson’s disease is a neurological disease with cardinal motor signs including bradykinesia and tremor. Although beta-band hypersynchrony in the cortico-basal ganglia network is thought to contribute to disease manifestation, the resulting effects on network connectivity are unclear. We examined local field potentials from a non-human primate across the naïve, mild, and moderate disease states (model was asymmetric, left-hemispheric dominant) and probed power spectral density as well as cortico-cortical and cortico-subthalamic connectivity using both coherence and Granger causality, which measure undirected and directed effective connectivity, respectively. Our network included the left subthalamic nucleus (L-STN), bilateral primary motor cortices (L-M1, R-M1), and bilateral premotor cortices (L-PMC, R-PMC). Results showed two distinct peaks (Peak A at 5–20 Hz, Peak B at 25–45 Hz) across all analyses. Power and coherence analyses showed widespread increases in power and connectivity in both the Peak A and Peak B bands with disease progression. For Granger causality, increases in Peak B connectivity and decreases in Peak A connectivity were associated with the disease. Induction of mild disease was associated with several changes in connectivity: (1) the cortico-subthalamic connectivity in the descending direction (L-PMC to L-STN) decreased in the Peak A range while the reciprocal, ascending connectivity (L-STN to L-PMC) increased in the Peak B range; this may play a role in generating beta-band hypersynchrony in the cortex, (2) both L-M1 to L-PMC and R-M1 to R-PMC causalities increased, which may either be compensatory or a pathologic effect of disease, and (3) a decrease in connectivity occurred from the R-PMC to R-M1. The only significant change seen between mild and moderate disease was increased right cortical connectivity, which may reflect compensation for the left-hemispheric dominant moderate disease state.

## Introduction

Parkinson’s disease (PD) is a progressive, neurodegenerative disorder, the cardinal motor signs of which may include akinesia, bradykinesia, and rest tremor. Its pathogenesis includes the death of dopaminergic neurons in the substantia nigra pars compacta; an area of the midbrain that is highly connected with the basal ganglia thalamocortical (BGTC) network and is important in the modulation and transmission of sensorimotor information (Beitz, 2014). Decades of research on PD support the involvement of widespread changes in neural activity within various nodal points of the BGTC network in motor sign manifestation (DeLong, 1990). Specifically, the onset of parkinsonism has been linked to excessively high spectral power in subthalamic nucleus (STN) local field potentials (LFPs) in the beta range (∼13–30 Hz), indicating that the disease causes this nucleus to become hyperactive (Brittain and Brown, 2014; Bore et al., 2020). More recently, however, greater emphasis has been given to understanding how the progressive loss of dopaminergic input alters connectivity across the various brain regions of the circuit, and, in turn, how such relationships are further altered as a function of therapeutic intervention and efficacy (Wu et al., 2012).

The STN is a key node in the basal ganglia (BG) circuitry (Galván et al., 2015). The basal ganglia receive glutamatergic excitatory cortical input through the striatum and STN. Information is then moved to the output nuclei of the BG, the internal globus pallidus (GPi) and substantia nigra pars reticulata (SNr). Noteworthy, projections from the striatum to the output nuclei are classified into the monosynaptic “direct” pathway, and the “indirect” pathway, a polysynaptic projection that traverses external globus pallidus (GPe) and STN then reaches the output nuclei. Moreover, the STN itself represents a third, important entry point for extrinsic cortical input to the BG network. This cortico-subthalamic route, along with its continuation to GPi/SNr has been referred to as the “hyperdirect” pathway since information flowing via this projection enters the BG output structures with a shorter delay than information transmitted along the direct and indirect corticostriatofugal pathways (Nambu et al., 2002).

Beyond nodal changes, a number of studies have explored the connectivity of the STN in parkinsonism in order to understand how disease onset and progression, and the corresponding increased STN beta power, change how the STN receives and transmits information to other key effectors of motor control. These other areas of interest include the basal ganglia nodes (Brown et al., 2001; Kahan et al., 2014), motor cortical regions (Hirschmann et al., 2013), the pedunculopontine nucleus (Li et al., 2016), and the cerebellum (Baggio et al., 2019). Because it is not currently fully understood the extent to which each of these areas within the sensorimotor circuit exhibit altered connectivity within the parkinsonian state and how this may lead to clinical manifestations of disease, study of electrophysiological connectivity between all of these regions is relevant. However, the STN and motor cortical areas are known to play a role in the motor symptoms of disease, and to our knowledge, their connectivity has not been explored in depth. Past studies have focused largely on the basal ganglia and only include the primary motor cortex to represent the motor cortical areas (Ghasemi and Mahloojifar, 2013) or focus only on the cortico-subthalamic connections (Williams et al., 2002) or the accompanying cortico-cortical connections (Hao et al., 2020), but not both. To date, only a handful of studies have focused on changes in the cortico-subthalamic network while also dividing the cortex into separate primary motor and premotor areas for each hemisphere (Baudrexel et al., 2011; Fernández-Seara et al., 2015; Kurani et al., 2015). Of these few studies, only Kurani et al. (2015) attempted to quantify changes in connectivity, derived in that case from fMRI, occurring across multiple levels of PD severity and against healthy controls.

In this study, we analyzed a cortico-subthalamic network consisting of the left subthalamic nucleus (L-STN), the right and left primary motor cortex (R-M1, L-M1) as well as the right and left premotor cortex (R-PMC, L-PMC) using Granger causality analysis (GCA). This technique provides an estimate of directed (bidirectional) network connectivity in both the time and frequency domains and is based on a linear multivariate autoregressive model. This network was examined across the naïve state, mild generalized parkinsonian state, and moderate left-hemisphere predominant parkinsonian state, demonstrating connectivity changes across model severity progression. This study used local field potentials (LFP) obtained from bilaterally implanted electrocorticography (ECoG) arrays and depth electrodes implanted in the region of the STN. We also analyzed power spectral density (PSD) and coherence to supplement the GCA results and provide further insights.

## Materials and Methods

All study-related procedures were performed in accordance with a protocol approved by the Institutional Animal Care and Use Committee of the Cleveland Clinic.

### Subject

**FIGURE 1:**
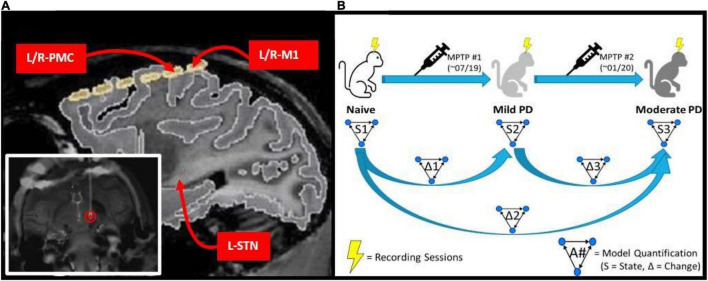
**(A)** MRI image of implanted electrode setup. Bottom left image shows the DBS lead implanted within the L-STN (red circle). Top right image shows cortical grid electrode with the bilateral PMC/M1 labeled, as well as the approximate location of the L-STN. **(B)** Experimental scheme which involves a progressive parkinsonian primate model with two sequential MPTP injections to induce first a symmetric mild disease state followed by a moderate, asymmetric, left-hemispheric dominant parkinsonian disease state. Multiple LFP recordings were taken in each state (Naïve, Mild PD, and Moderate PD). Granger causality analysis was performed on the recordings taken in each state (S1, S2, and S3), and then the differences between results were determined across states (Δ1, Δ2, and Δ3).

Data were acquired from a rhesus macaque non-human primate (14 y.o.; female). A custom-made six contact directional DBS lead (Abbott Medical) was implanted in the area of the left STN under stereotaxic guidance using methods described previously (Branco de Paiva et al., 2020). The six contacts of the DBS lead were split evenly between two rows, with each containing three contacts spanning ≈120 degrees circumferentially and spaced 0.08 mm apart. Each contact was 0.76 mm in length and the two rows were separated by 0.51 mm with the most distal row 0.51 mm from the tip of the lead. For the purpose of the current work, recordings from the three contacts of each row were pooled and differentially amplified, resulting in a single, adjacent bipolar signal from the STN region. Electrocorticographic recordings were made using a pair of bilaterally placed 12-contact, SureScan paddle electrodes (Medtronic; Minneapolis, MN) implanted subdurally through an open craniotomy. Each array spanned from the somatosensory cortex posteriorly to Brodmann area 46 (PF-A46) anteriorly. For the purpose of the current work, the two contact pairs overlying M1 were identified as those with the lowest threshold for motor activation when applying electrical stimulation to induce muscle twitch, which were further confirmed as best approximating the M1 region through coregistration of a pre-operative 7T MRI and post-operative CT scan. The PMC was defined as the next most anterior contact pair. The cortical channels were defined as the difference between two adjacent contacts which were both in the region of interest. The ends of the DBS electrode and ECoG arrays were routed to a chamber placed atop the head and secured using a combination of dental cement and surgical bone screws. A headpost (Gray Matter Research; Bozeman, MT) was further incorporated into the cranial implant over the occipital region of the skull to allow for head fixation and referencing during task performance and recording (Figure 1A). The mild parkinsonian state was induced by a series of three, daily intramuscular injections of the 1-methyl-4-phenyl-1,2,3,6-tetrahydropyridine (MPTP) neurotoxin (0.5, 0.2, and 0.3 mg/kg, respectively). The moderate state was introduced through the unilateral left intra-carotid infusion of MPTP (0.5 mg/kg; 1 mg/mL; 0.5 mL/min) using an endovascular approach (Bankiewicz et al., 1986) performed under fluoroscopic guidance. Bradykinesia scores were quantified across disease progression by quantifying the amount of time it took the subject to reach in front of them and tap a touch screen.

### Electrophysiology

Spontaneous LFP recordings were acquired with the awake animal secured in a custom primate chair with its head stabilized. Continuous direct or CCTV monitoring of the animal during each recording session was performed to ensure that the animal was alert with eyes open during data acquisition. Data were acquired using a Tucker Davis Technologies system, with an initial sampling rate of 24,414 Hz and using an anti-aliasing filter set at 45% of the sampling rate. Recording sessions were performed over a period of 3 months in the naïve state, with subsequent induction of the mild parkinsonian state as described above. After 5 months of recordings in the mild state, an additional month of recordings were made in the asymmetric, moderate parkinsonian state. This experimental scheme is illustrated in Figure 1B.

### Data Preprocessing

Data were bandpass filtered between 1 and 150 Hz (Butterworth, second order, two-pass), and a notch filter was applied to reduce 60 Hz line noise and its harmonics at 120 and 240 Hz (Barnett and Seth, 2011). The filtered signals were then downsampled to 400 Hz (Barnett and Seth, 2017). Thereafter, each session’s data were split into consecutive, non-overlapping 5-min (300 s) segments for GCA and 30 s segments for PSD and coherence analysis. We then normalized each LFP signal prior to running GCA to eliminate any bias in model estimation. All data preprocessing was performed using the FieldTrip toolbox (Oostenveld et al., 2011).

### Power and Coherence Analysis

PSD and coherence were calculated from the data, both to understand the general frequency content of the LFPs as well as to compare with GCA results for consistency. These analyses were carried out on each segment and then results were averaged across each experimental state. These computations were carried out using the FieldTrip toolbox (Oostenveld et al., 2011), which was structured inside of a custom-built MATLAB application.

### Granger Causality Analysis

Granger causality was calculated on a segment-by-segment basis, with results subsequently combined across all segments per experimental state. The multivariate granger causality (MVGC) toolbox (Barnett and Seth, 2014), structured inside a custom-built MATLAB application, was used to perform the formal GCA computation per segment and was also used for aspects of the compilation process.

### Granger Causality Definition and Theory

A detailed description of the mathematical theory of granger causality analysis (GCA), as well as how it has been adapted to a functional MATLAB toolbox, can be found in the main MVGC paper (Barnett and Seth, 2014), with further details on GCA theory found in Seth (2010). Granger causality (GC) is a method of quantifying “causality,” or directed statistical influence, between time series that is based on the multiple variant autoregression (MVAR) model (Granger, 1969; Geweke, 1982). In GC, if the past information of an observed time series x(*n*-k) can significantly improve the prediction of another time series y(*n*), then we can say that signal x(*n*) “Granger-causes” signal y(*n*). Thus, Granger causality provides insight into the “causal” interactions between structures, where the term “causal” means, approximately, the direction and magnitude of information flow (see Supplementary Material 1.1).

The first step in this process was the estimation of the model order which was done using the Bayesian information criterion (BIC) (Koehler and Murphree, 1988), and then with the selected model order, we computed the MVAR coefficients using the ordinary least squares (OLS) algorithm. After this, we calculated the autocovariance sequence as per the MVAR model. Eventually, GC in the frequency domain was calculated using the autocovariance sequence (Barnett and Seth, 2014).

### Modeling for Granger Causality Analysis

**FIGURE 2:**
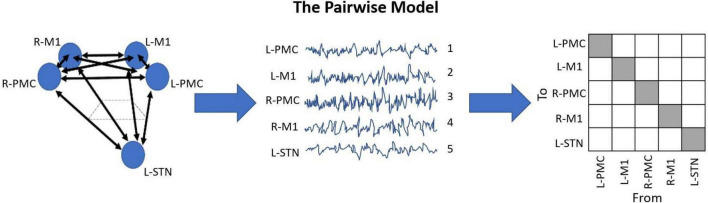
Diagram displaying the Pairwise Model. This model is the standard when it comes to G-causality computation, and simply analyzes the connections between each recorded node in the model. This is typically represented in an n-by-n matrix with indeterminate entries for the diagonals, where n is the number of unique channels in the model. The topographic diagram describing the model (left) is shown to be “recorded,” yielding the waveforms of each channel, ordered in an arbitrary manner (middle). Upon G-causality computation, this yields the adjacency matrix F (right). In this case, F happens to be a 5 by 5 matrix, as there are five channels. Note also how there are 20 unique causal entries (white squares), which is 5*(5-1).

As a result of our LFP recording setup, coupled with the nature of GCA, we developed a model for this analysis which we have been named the *Pairwise Model*. A detailed depiction of this model can be seen in Figure 2. The Pairwise Model (Figure 2) is the network of all directed interactions between each pair of individual recording sites in the experiment, which leads to 20 unique causal connections between the five nodes in the model (L-PMC, L-M1, R-PMC, R-M1, and L-STN).

### Granger Causality Metrics

Here, we describe the different Granger causality metrics which we have computed. First, the frequency domain GC was calculated, which is a spectrum. From this spectrum, average values were taken within certain frequency ranges to obtain three bandlimited GC metrics. The first, called the overall GC, was defined as the average GC between 0 and 50 Hz. We also determined *post hoc* that two major frequency domain Granger causality peaks emerged across the entire network, labeled Peak A (5–20 Hz) and Peak B (25–45 Hz) with absolute maxima of approximately 15 and 35 Hz, respectively. Peak A includes the theta, alpha, and low beta frequency ranges, and Peak B contains the high beta and low gamma frequency ranges. From these ranges, we have also computed bandlimited GCs as official metrics to be compiled. The average PSD and coherence within these bandlimited ranges were calculated as well, and statistics on these results were performed (the one difference is that the “overall” power and coherence were considered to be between 5 and 50 Hz instead of 0–50 Hz).

After computing all time and frequency domain Granger causalities for every trial in the dataset, it is necessary to average these values across each experimental state to obtain a summary result. The first major step of the compilation process is the filtering of trials against outliers, which helps to eliminate bias in the results from trials which may have been corrupted by noise such as movement artifacts (see Supplementary Material 1.2). Next, the important Granger causality metrics (frequency, time-domain, and bandlimited) are compiled from the raw trial data by way of computing the mean and standard error of each metric across all trials within each experimental state. All of the bandlimited metrics were computed from the frequency domain GCs for each trial before averaging.

### Statistics

Given the varying sample sizes across the different states as well as the clustered nature of the various trials in the data, a series of linear mixed-effects models were used to determine whether time-domain GC differed between states. A linear mixed-effects model is a flexible extension of linear regression that allows accurate modeling of multilevel, clustered data. The unit of analysis is a single GC value, and each GC value is nested within a recording session. The mixed-effects model accounts for the inherent correlation among observations (GC) from trials nested within each cluster (recording session).

Results are obtained as mean differences in GC after adjusting for clustering, which can then be hypothesis tested to produce *p*-values. Each model applied a compound symmetry covariance structure (equal variances and covariances) and each was individually evaluated for fit using the BIC (Koehler and Murphree, 1988) and visual examination of residuals. Analyses were two-tailed and were conducted on complete cases using SAS Studio v 3.7. *P*-values were adjusted using the Benjamini-Hochberg correction (Benjamini, 2010) for multiple comparisons under dependency. Adjusted *p*-values are compared to an alpha of 0.05 to indicate significance.

For power and coherence analysis, a simpler Mann-Whitney *U*-test was employed using GraphPad Prism version 8.0.0 for Windows, GraphPad Software, San Diego, California United States.^1^ This was done in order to find significant differences between states. The Benjamini-Hochberg correction for multiple comparisons at a significance level of 0.05 was used here as well. Because GraphPad Prism takes a maximum sample size of 512 samples, random samples of trials were taken from the naïve and mild states which had more trials than this.

## Results

A total of 15 LFP samples (averaging 46 min each) were recorded in the naïve state over a period of 5 months, while 16 samples (averaging 23 min each) were collected in the mild parkinsonian state over a period of 3 months and 5 samples (averaging 32 min each) were collected in the moderate parkinsonian state over a period of 1 month. These were further split into shorter segments (5 min for GCA; 30 s for power and coherence analysis) in duration before feature extraction. Overall, this led to the acquisition of 139, 75, and 32 segments for analysis of GCA in the naïve, mild, and moderate disease states, respectively. After outlier exclusion, this left approximately 90% of these sample sizes (125, 68, and 29 segments, respectively) to be averaged. For PSD and coherence analysis, there were 1,549, 758, and 329 segments to be averaged in the naïve, mild, and moderate disease states, respectively. No outliers were excluded as these sample sizes were much larger and presumably, more resistant to several outliers.

**FIGURE 3:**
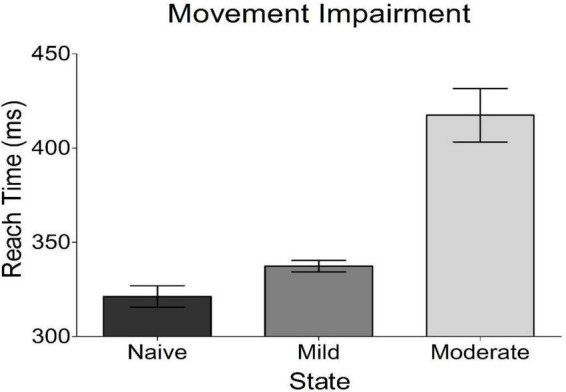
Behavioral results from reach time task. Plotted is the average time it took the subject to reach out and tap a touch screen placed in front of her. This is a measure of bradykinesia, a cardinal motor sign of parkinsonism. The subject got progressively worse with disease progression.

Bradykinesia was assessed in each of the three experimental states studied. We found that the subject’s task-related movement time increased with progression to both mild and moderate parkinsonism (Figure 3), with the degeneration between mild and moderate states being larger than that seen between naïve and mild states. Of note, both dopamine replacement and DBS therapies improved bradykinesia scores in this animal; these behavioral data have been reported previously in a related publication (Campbell et al., 2021).

### Power Spectral Density and Coherence

**FIGURE 4:**
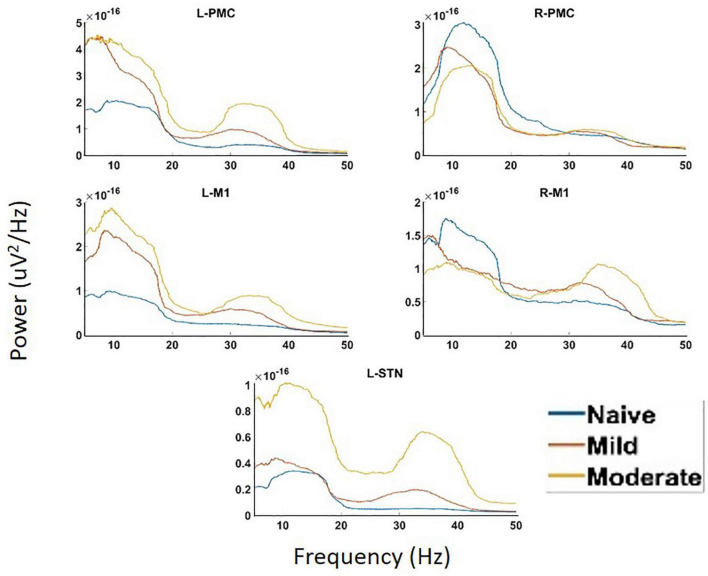
Power Spectral Density (PSD) calculated for all channels in the model of interest. There are two peaks present in the spectra, Peak A (5–20 Hz) and Peak B (25–45 Hz). There are significant increases in Peak A power in both the L-STN and left motor cortex and significant decreases in the right motor cortex after disease onset. There are significant increases in Peak B power everywhere except R-PMC after the induction of parkinsonism.

Analysis of PSD showed the presence of two peaks in the spectra, called Peak A (5–20 Hz) and Peak B (25–45 Hz) (Figure 4). These same two peaks were reproduced in both coherence and G-causality analyses. The amplitude of both of these peaks predominantly, but not ubiquitously, increased as the disease progressed. In particular, Peak A power increased in both of the left motor cortical regions (L-PMC, L-M1) as well as the L-STN, and decreased in the right motor cortical regions (R-PMC, R-M1). Peak B power increased monotonically across disease progression in all regions in the model except the R-PMC. In the R-PMC, there was a small decrease in power as the disease progressed from the naïve to mild state, and a larger increase between mild and moderate disease such that there was an overall increase in power. The changes in Peak B power for the R-PMC were also much smaller in magnitude than those of the other nodes in the model.

**FIGURE 5:**
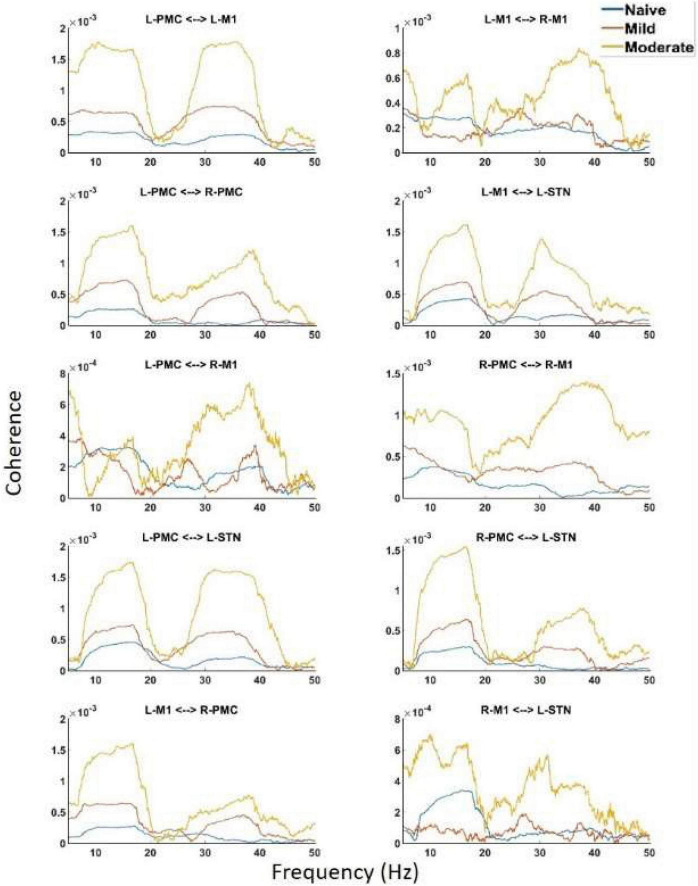
Coherence calculated for all channels in the model of interest. There are the same two peaks present which are seen in both PSD and G-causality. Widespread increases in both Peak A and Peak B are seen throughout the network across disease progression.

Undirected connectivity as measured by coherence also showed widespread increases in connectivity across both peaks as the disease progressed (Figure 5). In the Peak A range, these increases existed across most interactions but were not ubiquitous as the disease progressed from the naïve to the mild state (Peak A coherence between L-PMC and R-M1, L-M1, and R-M1, as well as R-M1 and L-STN were exceptions). However, Peak A coherence in the moderate state was increased relative to the naïve state across all interactions except for the L-PMC and R-M1 interaction. Peak B coherence increased across both state transitions for all interactions.

### Pairwise Model Granger Causality

**FIGURE 6:**
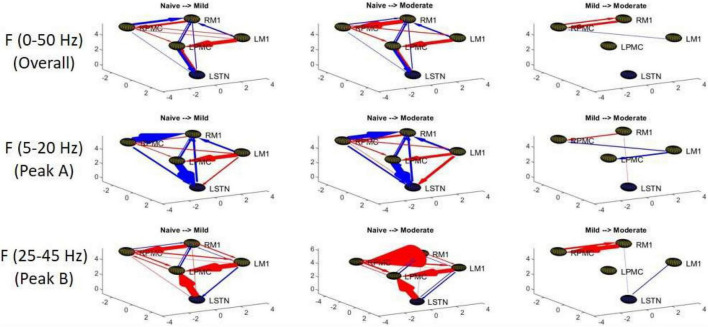
Topographic diagrams representing Granger causality analysis of the Pairwise Model. The topography of the model parallels that of Figure 2 and is taken from a left-coronal-sagittal viewpoint. Relative changes in interaction strength during state transitions are shown. Red represents an increase in G-causality and blue represents a decrease in G-causality. Note that only statistically significant connections are shown for both plots.

**FIGURE 7:**
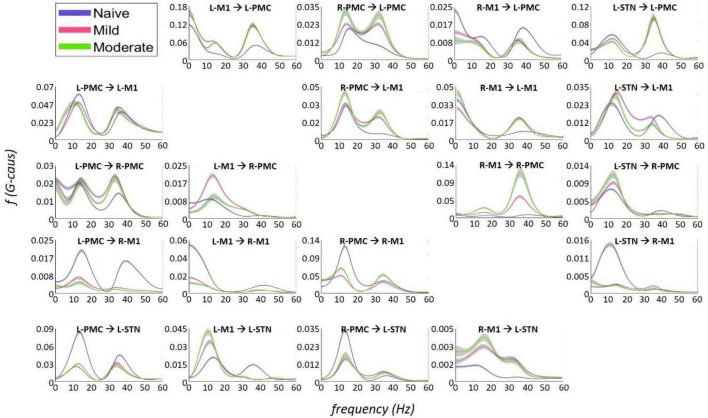
Frequency Domain Granger causality plots representing the Pairwise Model. The significant G-causal relationships between nodes can be seen to materialize almost entirely within the 0–50 Hz range, with two prominent peaks that have absolute maxima at ∼12–16 and ∼34–38 Hz. These peaks are referred to as “Peak A” and “Peak B,” respectively. The larger areas under these peaks are contained within the ranges of 5–20and 25–45 Hz, respectively. Note that each plot has its own y-axis limits, and so the magnitudes of the different interactions are not normalized to each other. Shaded bars are an estimate of the 95% confidence interval, calculated as 1.96 times the standard error of the mean.

Granger causality analysis showed several significant changes in both the cortico-subthalamic and cortico-cortical interactions in the pairwise network across the three model states (Figure 6). The primary cortico-subthalamic change in GC strength, which occurred between the naïve and mild parkinsonian state, was in the L-STN/L-PMC pair. This change in connectivity was reciprocal, with the L-PMC to L-STN interaction decreasing and the L-STN to L-PMC interaction increasing after disease onset. The reciprocity of the changes was segregated not only by direction but also by frequency range, with the L-PMC to L-STN decreasing in the range of Peak A (5–20 Hz) and the L-STN to L-PMC increasing in Peak B (25–45 Hz) (Figure 7). This trend of decreasing GC in Peak A and increasing GC in Peak B was common throughout the network. Additionally, a more modest decrease was seen from the L-STN to R-M1 in the Peak A range, and increasing GC was seen from the L-M1 to L-STN in the Peak A range as well. With respect to the progression of the disease state, these changes, like many in the network, primarily materialized during the onset of mild disease and remained relatively constant after the transition to the moderate state. Connectivity from the L-STN to ipsilateral cortical nodes (L-PMC, L-M1) exhibited a peak in the Peak B band whereas GC from the L-STN to contralateral cortical nodes (R-PMC, R-M1) did not. This implies that information flow from L-STN to motor cortex in the Peak B band may be lateralized to the ipsilateral side.

**FIGURE 8:**
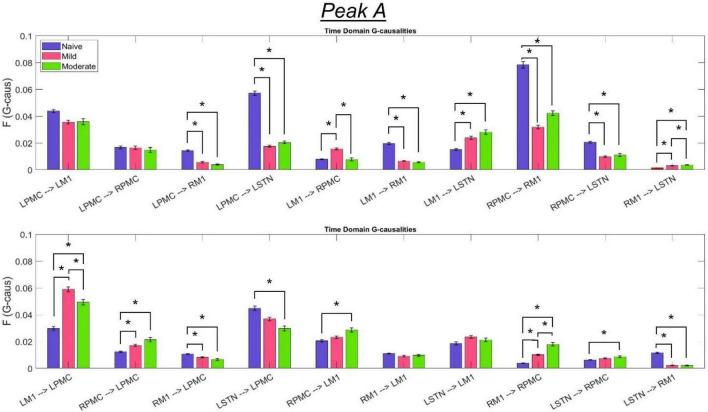
Bandlimited G-causalities in the Peak A (5–20 Hz) frequency range for the Pairwise Model. Note that the changes in these G-causalities were dominated by large decreases after the induction of parkinsonism. Error bars represent the standard error of the mean. Bright red bars represent non-significant G-causalities in a single experimental state, and asterisks represent significant differences between G-causalities in different experimental states at a significance level of α = 0.05. Note that this figure has the same *y*-axis limit as Figure 9 which presents the Peak B G-causalities, such that the magnitudes of G-causalities across the frequency ranges can be more easily compared.

**FIGURE 9:**
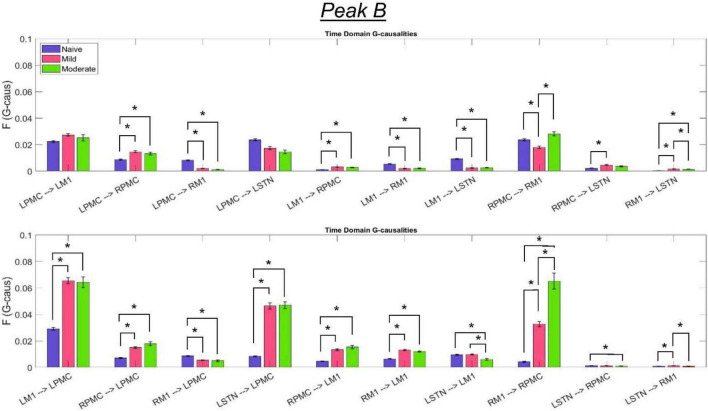
Bandlimited G-causalities in the Peak B (25–45 Hz) frequency range for the Pairwise Model. Note that the changes in these G-causalities were dominated by large increases after disease onset. Error bars represent the standard error of the mean. Bright red bars represent non-significant G-causalities in a single experimental state, and asterisks represent significant differences between G-causalities in different experimental states at a significance level of α = 0.05. Note that this figure has the same y-axis limit as Figure 8 which presents the Peak A G-causalities, such that the magnitudes of G-causalities across the frequency ranges can be more easily compared.

The most salient cortico-cortical changes were significant increases in the strength of GC in the direction of M1 to ipsilateral PMC, bilaterally (L-M1 to L-PMC and R-M1 to R-PMC). These increases were concentrated in the Peak B band. The left hemispheric interaction, L-M1 to L-PMC, only increased in amplitude during the onset of mild disease, whereas the right hemispheric interaction, R-M1 to R-PMC, increased throughout the entire progression of disease. This caused the net change of the R-M1 to R-PMC interaction to outweigh that of its corresponding left hemispheric counterpart. Another salient change in the cortico-cortical interactions of the model was that there was a decrease in causality from the PMC to the M1 on the right side (R-PMC to R-M1), forming a reciprocal relationship with the increasing R-M1 to R-PMC interaction mentioned above. This decrease in causality occurs primarily in the Peak A range during the naïve to mild transition and is partially reversed during the mild to moderate transition (Figure 8). Both of the left hemispheric cortical nodes also exhibit decreasing causal effects on the R-M1 within the Peak A range. This occurs almost entirely during the onset of mild disease and may be associated with the decreasing power observed in the R-M1 in the Peak A range. Finally, modest increases in GC from the R-PMC to both nodes on the left hemisphere were found to accumulate across the entire disease progression, mainly in the Peak B band (Figure 9).

### Statistical Results for Analyses

The following tables contain the adjusted *p*-values obtained from the statistical analyses associated with each of the two connectivity analyses performed above (Table 1: Power and Coherence Analysis and Table 2: Granger Causality Analysis). Each *p*-value is compared to a significance level of α = 0.05 and has been corrected for multiple hypotheses. Statistics are shown only for the changes between experimental states (see section “Statistics”). The tables are color-coded such that a red cell denotes a significant increase, and a blue cell denotes a significant decrease.

**TABLE 1:** Adjusted *p*-values for Mann–Whitney *U*-test, pairwise model power, and coherence analysis (bold, colored is significant at α = 0.05).

Red = Increase Blue = Decrease	Overall (5–50 Hz)			Peak A (5–20 Hz)			Peak B (25–45 Hz)		
	Naïve to Mild	Naïve to moderate	Mild to moderate	Naïve to Mild	Naïve to moderate	Mild to moderate	Naïve to Mild	Naïve to moderate	Mild to moderate
**Power spectral density**									
L-PMC	**0.0013**	**0.0344**	**0.0001**	**<0.0001**	0.2729	**<0.0001**	0.1486	**0.0002**	**0.0002**
L-M1	0.1011	**0.0004**	**0.0002**	**0.0447**	0.0802	**0.0017**	**0.0035**	**0.0010**	**<0.0001**
R-PMC	**<0.0001**	**<0.0001**	**<0.0001**	**<0.0001**	**<0.0001**	**<0.0001**	**<0.0001**	**<0.0001**	**<0.0001**
R-M1	**<0.0001**	**<0.0001**	0.1753	**<0.0001**	**<0.0001**	**0.0193**	**<0.0001**	**<0.0001**	**<0.0001**
L-STN	**<0.0001**	**<0.0001**	**<0.0001**	**<0.0001**	**0.0480**	**<0.0001**	**<0.0001**	**<0.0001**	**0.0274**
**Coherence**									
L-PMC and L-M1	**<0.0001**	**<0.0001**	0.1098	**<0.0001**	**0.0157**	**<0.0001**	**<0.0001**	**<0.0001**	0.1121
L-PMC and R-PMC	**<0.0001**	**<0.0001**	0.7377	**<0.0001**	**0.0266**	**0.0097**	**<0.0001**	**<0.0001**	0.0552
L-PMC and R-M1	**<0.0001**	**<0.0001**	**0.0169**	**<0.0001**	**<0.0001**	**<0.0001**	**<0.0001**	**<0.0001**	0.0905
L-PMC and L-STN	**<0.0001**	**<0.0001**	0.1098	**<0.0001**	**<0.0001**	0.5648	**<0.0001**	**<0.0001**	0.2826
L-M1 and R-PMC	**<0.0001**	**<0.0001**	0.7226	**<0.0001**	**0.0074**	**<0.0001**	**<0.0001**	**<0.0001**	**<0.0001**
L-M1 and R-M1	**<0.0001**	**<0.0001**	0.0761	**<0.0001**	**<0.0001**	**<0.0001**	**<0.0001**	**<0.0001**	**0.0161**
L-M1 and L-STN	**0.0062**	0.0903	0.1098	**0.0009**	**0.0002**	0.5205	**0.0142**	0.0855	0.0905
R-PMC and R-M1	**<0.0001**	**<0.0001**	**<0.0001**	**<0.0001**	**<0.0001**	0.5719	**<0.0001**	**<0.0001**	**<0.0001**
R-PMC and L-STN	**<0.0001**	**<0.0001**	0.1255	**<0.0001**	**<0.0001**	0.5321	**<0.0001**	**<0.0001**	0.0986
R-M1 and L-STN	**<0.0001**	**<0.0001**	0.1098	**<0.0001**	**<0.0001**	0.5283	**<0.0001**	**<0.0001**	0.0986

**TABLE 2:** Adjusted *p*-values, Pairwise Model Granger causality analysis (bold, colored is significant at α = 0.05).

Red = Increase Blue = Decrease	Overall (0–50 Hz)			Peak A (5–20 Hz)			Peak B (25–45 Hz)		
	Naïve to Mild	Naïve to moderate	Mild to moderate	Naïve to Mild	Naïve to moderate	Mild to moderate	Naïve to Mild	Naïve to moderate	Mild to moderate
L-PMC to L-M1	0.9017	0.9017	0.8687	0.1195	0.2130	0.9059	0.0761	0.5435	0.5931
L-M1 to L-PMC	**0.0002**	**0.0002**	0.3307	**0.0003**	**0.0003**	**0.0174**	**0.0003**	**0.0003**	0.6944
L-PMC to R-PMC	0.0775	0.7716	0.3705	0.9527	0.5322	0.5144	**0.0005**	**0.0187**	0.8713
R-PMC to L-PMC	**0.0002**	**0.0002**	0.0736	**0.0048**	**0.0003**	0.0898	**0.0003**	**0.0003**	0.0499
L-PMC to R-M1	**0.0002**	**0.0002**	0.2961	**0.0003**	**0.0003**	0.4180	**0.0003**	**0.0003**	0.4763
R-M1 to L-PMC	**0.0002**	**0.0002**	0.3708	**0.0377**	**0.0144**	0.3428	**0.0007**	**0.0043**	0.6944
L-PMC to L-STN	**0.0002**	**0.0002**	0.7727	**0.0003**	**0.0003**	0.6080	0.2426	0.0485	0.2776
L-STN to L-PMC	**0.0002**	**0.0030**	0.4619	0.0698	**0.0157**	0.2875	**0.0003**	**0.0003**	0.9107
L-M1 to R-PMC	**0.0002**	0.6319	**0.0036**	**0.0003**	0.9566	**0.0046**	**0.0003**	**0.0005**	0.5931
R-PMC to L-M1	**0.0002**	**0.0002**	0.2087	0.1566	**0.0078**	0.1048	**0.0003**	**0.0003**	0.3437
L-M1 to R-M1	**0.0002**	**0.0002**	0.3427	**0.0003**	**0.0003**	0.4190	**0.0003**	**0.0003**	0.9107
R-M1 to L-M1	0.1350	0.2397	0.9017	0.2591	0.3428	0.9059	**0.0003**	**0.0003**	0.4899
L-M1 to L-STN	0.6740	0.2397	0.1350	**0.0048**	**0.0003**	0.0734	**0.0003**	**0.0003**	0.9312
L-STN to L-M1	0.1242	0.5918	0.0965	0.1017	0.5536	0.5558	0.9312	**0.0093**	**0.0092**
R-PMC to R-M1	**0.0002**	**0.0106**	**0.0043**	**0.0003**	**0.0003**	0.1030	**0.0182**	0.0744	**0.0009**
R-M1 to R-PMC	**0.0002**	**0.0002**	**0.0002**	**0.0003**	**0.0003**	**0.0003**	**0.0003**	**0.0003**	**0.0003**
R-PMC to L-STN	**0.0002**	**0.0026**	0.9017	**0.0003**	**0.0003**	0.5014	**0.0003**	0.0548	0.1070
L-STN to R-PMC	0.1722	0.0544	0.3705	0.2034	**0.0302**	0.2279	0.1917	**0.0393**	0.3039
R-M1 to L-STN	**0.0002**	**0.0002**	0.0495	**0.0070**	**0.0003**	**0.0098**	**0.0003**	**0.0003**	**0.0410**
L-STN to R-M1	**0.0002**	**0.0002**	0.7716	**0.0003**	**0.0003**	0.9059	**0.0079**	0.5931	**0.0154**

## Discussion

### Pathological Connectivity Predominant Within High Beta and Low Gamma Ranges

In the parkinsonian primate model, connectivity within the cortico-subthalamic motor network appears to be predominant within two select frequency ranges, both of which include the beta band. The connection strength as measured by G-causality in the higher, Peak B, range increased after the introduction of the disease while the lower, Peak A, range magnitude decreased, such that high beta and low gamma connectivity was dominant in the parkinsonian animal. Connection strength as measured by coherence increased across both peaks. The presence of beta-band hyper-synchronization within the cortico-basal ganglia motor loops is a hallmark of PD and has been reported by many studies (Brown et al., 2001; Fogelson et al., 2006; Avila et al., 2010). However, the specific range of frequencies reported as “beta-band” varies, and usually a low beta (Mallet et al., 2008; Eusebio et al., 2009) or high beta (Sharott et al., 2005; Ramirez Pasos et al., 2019) range with similar frequency bands to the peaks found here, or both (Williams et al., 2002; Fogelson et al., 2006; Avila et al., 2010), are reported. Both of these beta ranges likely exhibit peaks in at least some human and primate subjects, but it is less clear if there is more than one frequency peak in parkinsonian rodents, where power and connectivity analyses have mainly been shown to produce a single peak in the 10–30 Hz range (Li et al., 2016; West et al., 2018). Also, the finding that the Peak B (high beta/low gamma) range dominated the Peak A (theta/alpha/low beta) range in terms of absolute magnitude of G-causality is corroborated by several studies, all of which report either coherence or directed connectivity spectrum that peaks between 20 and 40 Hz (Williams et al., 2002; Fogelson et al., 2006; Li et al., 2016; Ramirez Pasos et al., 2019). However, a recent study in four vervet monkeys contradicts this, finding the dominant frequency range for both power and coherence in the parkinsonian animal to be 8–24 Hz (Iskhakova et al., 2021). As our study was conducted in a rhesus macaque, this difference could be related to species or could simply vary between individual cases (as both studies employed small sample sizes and there was variance in dominant frequency within the study conducted by Iskhakova et al., 2021). It is also interesting to note that the frequency range of increased power and increased connectivity need not be the same; Litvak et al. (2011) found that STN power peaked at 15–20 Hz whereas STN-cortex coherence peaked between 20 and 30 Hz. Our study partially corroborates this by finding that the power is maximal in the Peak A range but that, for certain interactions, GC and coherence are maximized in the Peak B range. In this work, we have extended the findings reported previously not only by characterizing the frequency content of electrophysiology between cortex and STN, but by also characterizing the magnitude and directionality of connectivity between key nodes in the cortico-subthalamic network.

### Cortico-Subthalamic Changes in the Pathological Beta Range

We observed significant changes in GC between the L-PMC and L-STN associated with the onset of parkinsonism. These changes were reciprocal in nature, in the sense that the onset of disease caused the cortex-driven interaction to decrease in the Peak A band (5–20 Hz) and the STN-driven interaction to increase in the Peak B band (25–45 Hz). The existence of a strong cortico-subthalamic coupling which intensifies after the development of PD (Baudrexel et al., 2011; Fernández-Seara et al., 2015; Kurani et al., 2015) may partially be reflective of a pathological increase in the hyperdirect pathway (Baudrexel et al., 2011). Notably, Baudrexel et al. (2011) conducted an fMRI study in off-medicated PD patients that showed increased functional connectivity between the STN and M1, as well as the midline premotor and supplementary motor areas. Similarly, Kurani et al. (2015) used fMRI to characterize changes across two parkinsonian states, *de novo* (a mild, medication-naïve disease state) and moderate. The authors observed a strengthened interaction between motor cortical areas and STN, with these increases found to be most pronounced for interactions between the STN on the more-affected side and both cortical hemispheres (Kurani et al., 2015). Similar to our findings, increased connectivity was mainly concentrated in the premotor areas. Also in agreement was their finding that the connectivity values obtained for both severity groups did not differ significantly, with both exhibiting the changes described relative to healthy controls. This suggests that pathologic connectivity changes occur primarily during the onset of mild disease and do not markedly change throughout disease progression. Notably, an fMRI study conducted by Fernández-Seara et al. (2015) also demonstrated an increased STN-motor connectivity across both primary motor and premotor areas; however, those authors found significant changes from bilateral STNs to the left hemisphere.

In terms of directionality, our results showed that in the naïve state, cortex-led connectivity is predominant, but that the induction of parkinsonism led to a decrease in cortex-led connectivity and an increase in STN-led connectivity such that the connection in the diseased state was predominantly STN-driven. This effect was mainly due to the changes seen between L-STN and L-PMC in particular. Our result that the STN led cortex in the parkinsonian state is supported by a study conducted by Li et al. (2016) which examined LFP connectivity between the STN, M1, and the pedunculopontine nucleus (PPN) of ambulating parkinsonian rats. Using GC, the authors found a significant increase in connectivity in the STN to M1 direction after the induction of the parkinsonian state, which was further shown to be attenuated by levodopa treatment. Another study investigating parkinsonian rats also found an increase in STN to M2 (secondary motor cortex) connectivity, even within the context where M2 to STN connectivity was dominant overall (West et al., 2018). It has been shown previously that the M1 increasingly drives STN after the induction of PD (Williams et al., 2002; Fogelson et al., 2006), and that this is not modulated by dopaminergic medication (Litvak et al., 2011). While this result may seem to contradict our finding of decreased cortical drive of STN in PD, we did find that the M1 in particular increased in GC influence on the STN in the Peak A band. Therefore, it is possible that in PD the STN increasingly drives PMC while itself being more influenced by M1 and other cortical areas.

### Cortico-Cortical Beta-Band Connectivity Changes in Parkinsonism

There is some degree of consensus that, in PD, the supplementary motor area (SMA) and medial premotor areas are inhibited while the lateral PMC and M1 become hyperactivated bilaterally (Sabatini et al., 2000; Haslinger et al., 2001; Wu and Hallett, 2005; Wu et al., 2009a,b) relative to healthy controls. In our study, the M1 is also seen to bilaterally drive its ipsilateral PMC at an increased level of connectivity, which could contribute to a bilateral increase in PMC activation. These findings suggest that the premotor areas may play a role in selective motor inhibition and tailoring of outputs in the disease state.

Several studies have asked whether PD leads to an overall increase or decrease of motor cortical connectivity. Silberstein et al. (2005) used transformed coherence to compare the strength of cortico-cortical connectivity between different EEG electrodes across PD patients and healthy controls. The authors found that the strength of connectivity in the low beta (10–18 Hz) and high beta (23–32 Hz) bands was positively correlated with the severity of motor impairment, as obtained by UPDRS motor scores. This effect was especially pronounced within the low beta band, and within this band, the correlation was predominantly exhibited within the central cortical areas (areas that approximate PMC and M1). Further, the study found that administration of levodopa or therapeutic STN DBS led to a negative correlation between the change in cortico-cortical coherence and UPDRS III score, such that the greater the reduction in coherence, the greater the clinical improvement. This suggests that motor cortical regions may become more connected to each other while becoming less connected to certain basal ganglia regions.

### Hemispheric Changes in Relation to the Asymmetry of the Moderate Disease State

One last element of our study which is worth noting is that the connectivity changes which were seen during the mild to moderate PD transition need to be considered in lieu of the asymmetric, left hemispheric dominant nature of the induced moderate disease state. In other words, although in the mild state both hemispheres would be expected to be equally affected by the disease process, the left intra-carotid nature of the second injection should cause the moderate disease state to be more skewed toward dysfunction in the left hemisphere.

When considered with reference to this disease model, one may be led to hypothesize that only GC interactions involving the left hemisphere would be exacerbated during the mild to moderate transition and the right hemispheric interactions would be left unperturbed. Our results contradict this hypothesis, instead of finding that the GC changes seen in the left hemisphere are small when compared to those in the right hemispheric cortex. We speculate that perhaps there was indeed further network degeneration on the left side which did not manifest as altered connectivity via the GC metric, and that the increased GC in the right cortical regions is a compensatory reaction to this undetected degeneration in the left hemisphere. Indeed, Kurani et al. (2015) found that although both mild and moderate PD patients had similar cortico-subthalamic connectivity profiles on the more affected side, there were significant symptomatic differences between the two groups. It is also known that PD not only alters the connectivity profile of the BGTC circuit but also changes the nature of these interactions via firing patterns (Rubin et al., 2012). Therefore, it is entirely possible that the transition from mild to moderate disease could be accompanied by altered BGTC circuit communication on the more affected side not in the form of altered connectivity strength but instead in the form of altered communication patterns. These putative changes could lead to reactionary changes in connectivity on the less affected side, such as those we have detected here.

## Study Limitations

Some limitations in current work will need to be considered in future studies. Notwithstanding, a limitation of this present work that will need to be taken into consideration in future studies is the limited statistical power in macaque studies (Wakabayashi et al., 2018), this may be complemented by using larger-sample sizes in the primate model or by collecting local field potentials (LFPs) in patients undergoing DBS surgery. This study in particular only analyzed data from a single primate subject, whereas many primate studies try to reproduce findings in a second primate subject. The absence of a second subject limits the ability of these findings to be generalized. Additionally, recordings from both subthalamic nuclei would eliminate interpretation bias toward the left hemisphere and would also allow cortical changes to be assessed in terms of their connections to the R-STN as well as to the L-STN. Further, induction of the moderate state was biased toward predominantly impacting the left hemisphere, so studies which either induce a symmetric or right hemispheric dominant moderate parkinsonian state would help to complement this one by determining any differential effects on network connectivity.

## Conclusion

We found a consistent pattern of changes in network connectivity between the healthy and disease states. These were restricted mostly to two frequency ranges which include the beta band and are typically considered to be implicated in the pathology of the disease. Notably, an increased amount of high beta/low gamma transmission was seen from the L-STN to its ipsilateral PMC, which may be related to a pathological hyperactivity within the STN emanating to the cortex (Oswal et al., 2016). Additionally, there was increased transmission from both motor cortices to their ipsilateral premotor cortices in the high beta/low gamma band, which may be reflective of either motor compensation or pathological “locking” via hyper-synchronization. Overall, the network increased in both power and coherence, which may also be reflective of excessive connectivity in the beta band. Future directions involve the analysis of how STN DBS impacts this network.

## Data Availability Statement

The raw data supporting the conclusions of this article will be made available by the authors, without undue reservation.

## Ethics Statement

The animal study was reviewed and approved by the Institutional Animal Care and Use Committee of the Cleveland Clinic.

## Author Contributions

AM and KB conceived and designed the experiments. BC, HC, and FP performed the experiments. JB, CT, and OH analyzed the dataset. JB and CT wrote the manuscript. All authors reviewed the manuscript.

## Conflict of Interest

AM and KB had potential financial conflict of interest with this research related to intellectual property or consulting and distribution rights in Enspire DBS. AM was a consultant to Abbott and Cleveland Clinic receives fellowship support from Medtronic. The Cleveland Clinic Conflict of Interest (COI) committee has approved a plan for managing these conflicts of interest. The authors have adhered to the management plan in the conduct and reporting of research findings. None of these entities had any role in the research or preparation of the manuscript. The remaining authors declare that the research was conducted in the absence of any commercial or financial relationships that could be construed as a potential conflict of interest.

## Publisher’s Note

All claims expressed in this article are solely those of the authors and do not necessarily represent those of their affiliated organizations, or those of the publisher, the editors and the reviewers. Any product that may be evaluated in this article, or claim that may be made by its manufacturer, is not guaranteed or endorsed by the publisher.

## Footnotes

1
www.graphpad.com
