# Supplementary material for: Consistent Changes in Cortico-Subthalamic Directed Connectivity Are Associated With the Induction of Parkinsonism in a Chronically Recorded Non-human Primate Model
Source: Front Neurosci. 2022 Mar 4;16:831055. doi: 10.3389/fnins.2022.831055 (PMC8930827; doi:10.3389/fnins.2022.831055)
Supplement: Supplementary file 1 [file Data_Sheet_1.docx]

Supplementary Material

# Materials and Methods

## A Note on the Meaning of Granger Causality

Put simply, Granger causality provides a measure of the quantified magnitude of the directed “causal” relationship between two nodes in a dynamic network, although it is generally not seen as “causal” in a formal or structural sense and is seen more as a metric of functional connectivity (Barnett et. al., 2009). Therefore, it is helpful to define what the term “causality” actually means when applied to Granger causality as a measure of directional connectivity. In terms of the actual meaning of this “causal” relationship, Granger causality has been proven mathematically equivalent to the information-theoretic measure of transfer entropy, which essentially measures information transfer between two nodes, in the special case of Gaussian-distributed input data (Barnett et. al., 2009). Now, the concern has been raised (and appropriately so) that neural data contains nonlinearities which prevent it from being Gaussian-distributed, and so this equivalence indeed does not hold (Lindner et. al., 2011). The analogue of the magnitude of Granger causality to the strength of information transfer between nodes, however, is still a useful concept, as in this case GCA simply quantifies this transfer, or “causality”, based on only the linear relationships in the data. Still, metrics such as transfer entropy have aroused interest in the literature in recent years as they do not suffer from the same limitations as GCA: namely, there is no reliance on a model of the data, and the measure captures nonlinearities in the data (Lindner et. al., 2011). However, many of these measures are less mathematically developed than GCA, as, for example, transfer entropy was originally formulated in 2000 (Schreiber 2000) and has only recently been extended to the frequency domain (Chen et. al., 2019; Pinzuti et. al., 2020). Last, it appears that Granger causality itself has been generalized to the nonlinear case, although this technique does not seem to be widely employed in neuroscience literature (Marinazzo et. al., 2008).

## Outlier Detection for Granger Causality Result Compilation

One challenge associated with averaging the GC values associated with each trial to obtain a summary result was the filtering out of outlier trials for each experimental state (see **Section 2.7** of the main paper). Outlier results could have been caused by a large amount of noise in the electrophysiology of certain trials, such as from movement artifacts or environmental noise. Therefore, it was important to have a procedure in place to exclude potential outlier trials which could bias the results.

To find and exclude outliers, the frequency domain G-causality for each trial (which is a vector) was sampled at random points in the frequency range of interest (0-50 Hz) across trials. The mean value of all trials at each sampled frequency point was then compared to the values for each trial using a single metric called the Mahalanobis distance (which is, roughly, a multidimensional generalization of the variance) (Li et. al., 2019). The trials which have the top 10% of values for the Mahalanobis distance were excluded as outliers. This procedure is sensitive not only to variance from the mean in terms of overall GC amplitude, but also to variance with respect to frequency content, and was chosen to detect outliers for this reason.

## Combined Model for Granger Causality Analysis


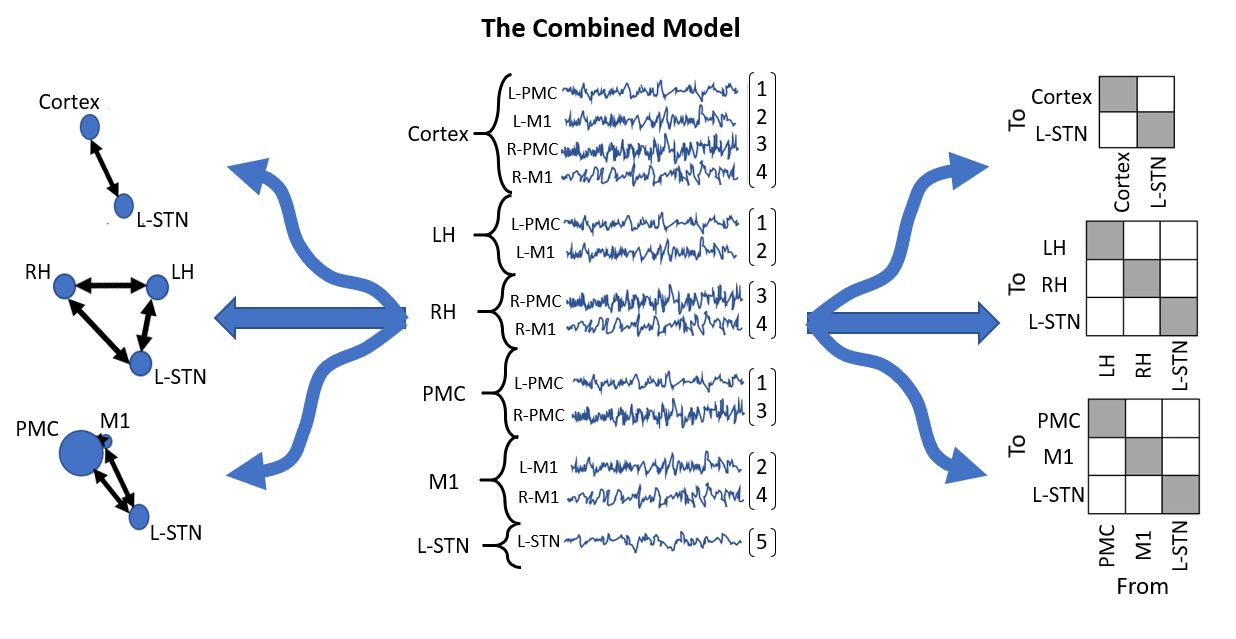
In addition to the Pairwise Model employed within the main publication, we also analyzed an alternative model called the Combined Model **(Figure S1)**. This model is formed by creating relevant groups of recording channels which comprise larger brain regions and analyzing all possible interactions between these groups which do not include overlapping channels. This leads to three possible network structures in this overall Combined Model. The Pairwise Model gives a picture of the model with the most granularity, but the Combined Model is better at summarizing the general model content and giving immediate insight into overall trends. Considering the relatively few channels of interest, the Combined Model does not yield many unique insights beyond those already given by the Pairwise Model. Nevertheless, we have included these results in this Supplementary Material (see **Suppl. 2.2 & 2.3**) as a methodological suggestion to support future GCA endeavors with larger amounts of recorded channels in the model of interest. For example, if G-causality is to be analyzed on an EEG network of 32 channels, the resulting 32*(32-1) = 992 unique GC interactions could be potentially messy and confusing to understand. By instead breaking the channels up into several macroscopic groups (e.g. motor, associative, occipital, etc.) and then analyzing GC between these, meaningful and more easily digestible trends might be seen that would be more easily missed in the conventional, pairwise network.

**Figure S1.** Combined Model for G-causality Analysis. This combined model allows for multivariate channel groups, which correspond to macroscopic brain regions, to be defined and for corresponding G-causalities to be calculated between these macroscopic regions (and their channel groups). Notice how multiple causal network models stem from this one Combined Model. This has to do with the overlap in channels. The number of unique directed interactions in each (sub)-network is the number of white squares in its adjacency matrix (right). Topographic diagrams (left) remain in coronal view.

# Results

## Pairwise Model Granger Causality


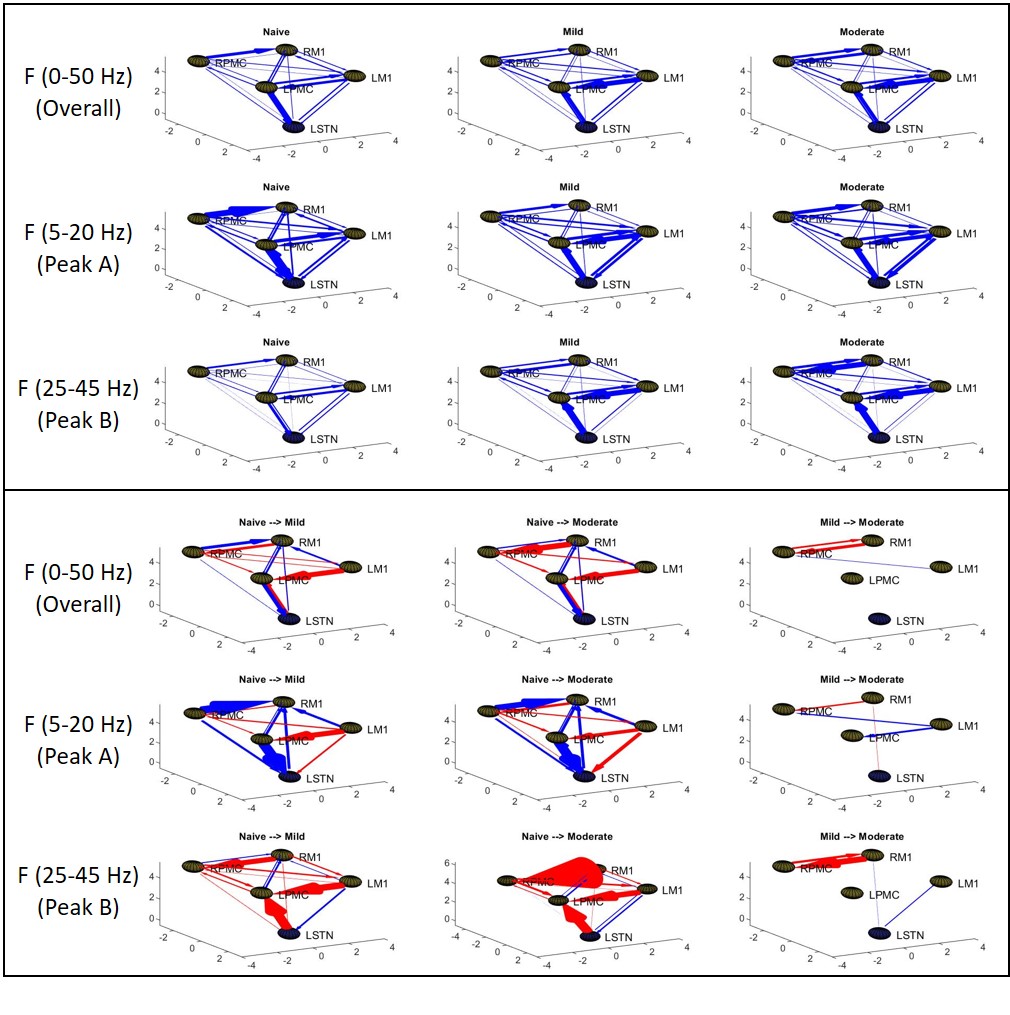


**(A)**

**(B)**

F**igure S2.** Topographic diagrams representing Granger causality analysis of the Pairwise Model. The topography of the model parallels that of Figures 1 and 2 and is taken from a left-coronal-sagittal viewpoint. **(A)** Relative GC interaction strength across the three experimental states. Arrow thickness represents the strength of directed connectivity. **(B)** Relative change in interaction strength during state transition. Red represents an increase in G-causality and blue represents a decrease in G-causality. Note that only statistically significant connections are shown for both plots. Also note that **Section B** of this figure is identical to **Figure 4** of the main paper.


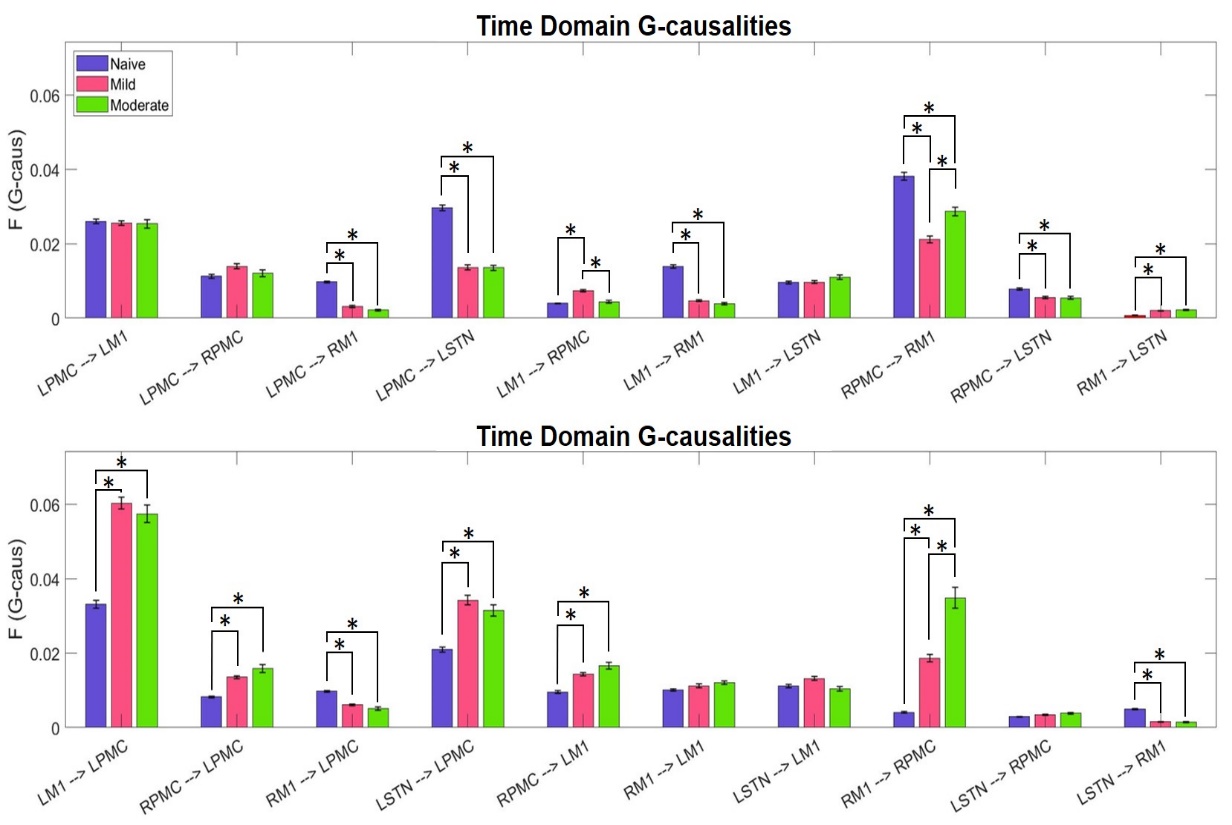
 Below is a figure which shows the overall Granger causality values (0-50 Hz) for the Pairwise Model.

**Figure S3.** Time Domain G-causalities for the Pairwise Model. Overall G-causalities between 0-50 Hz. Note that these causalities are referenced to a common y-axis limit and so the magnitudes across interactions are relative to each other. Error bars represent standard error of the mean, and asterisks represent statistical significance at α = 0.05.

Below is a figure which shows the values of the bandlimited Granger causality metrics (Peak A; 5-20 Hz and Peak B; 25-40 Hz) for all interactions in the Pairwise Model, along with their statistical significance.


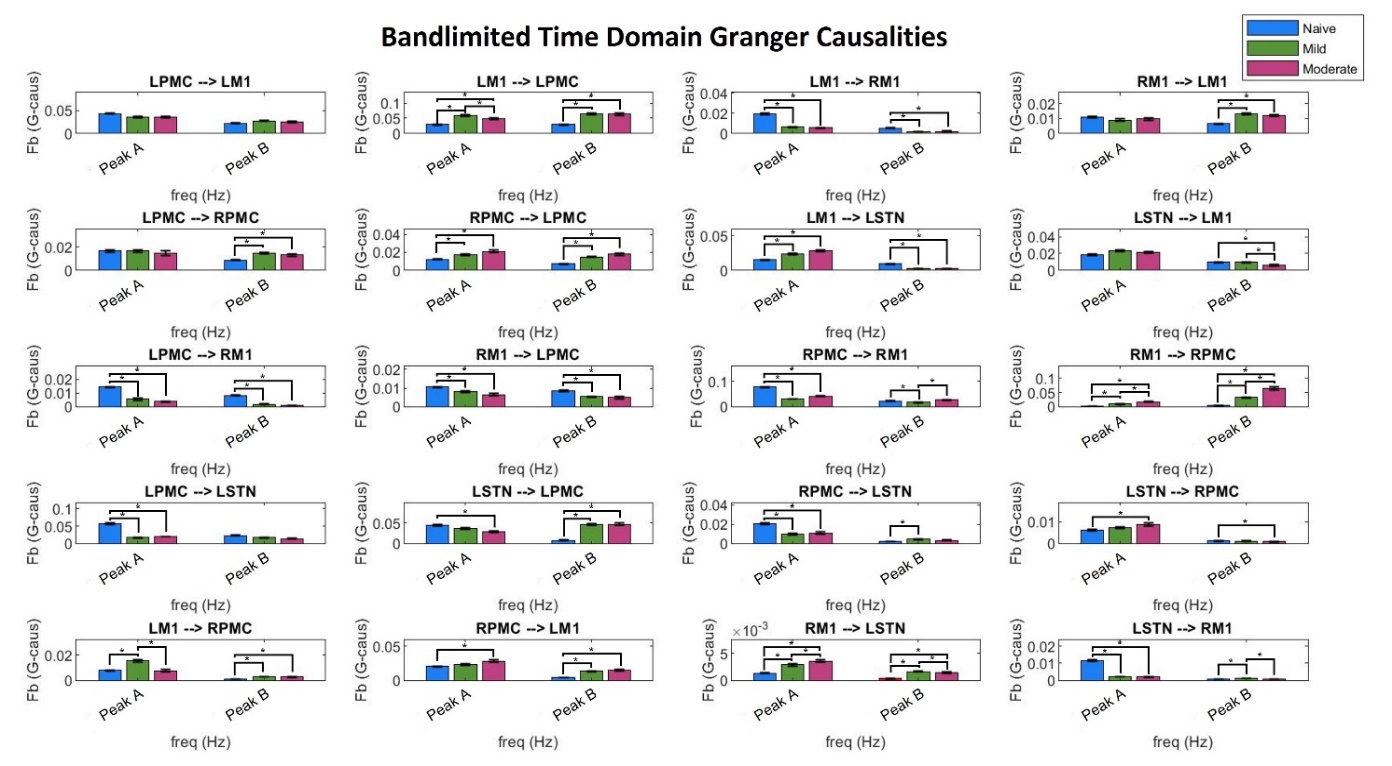


**Figure S4.** Bandlimited G-causalities for the Pairwise Model. The Peak A (5-20 Hz) G-causalities mainly decreased after the induction of Parkinsonism, and the Peak B (25-40 Hz) G-causalities mainly increased after disease onset. Error bars represent standard error of the mean. Asterisks represent significant differences between G-causalities in different experimental states at a significance level of α = 0.05. Note that this figure presents the same data as **Figures 6 and 7** of the main paper, but does not reference different interactions to a common y-axis limit which means that magnitudes across interactions cannot be easily compared. Instead, this plot presents the G-causality values for both frequency ranges for a given interaction within the same panel with the same y-axis limit, so that the relative magnitudes across frequency range can be more easily compared.

## Combined Model Granger Causality

Below are the figures associated with the Combined Model G-causality analysis. A description of the results will be presented after.


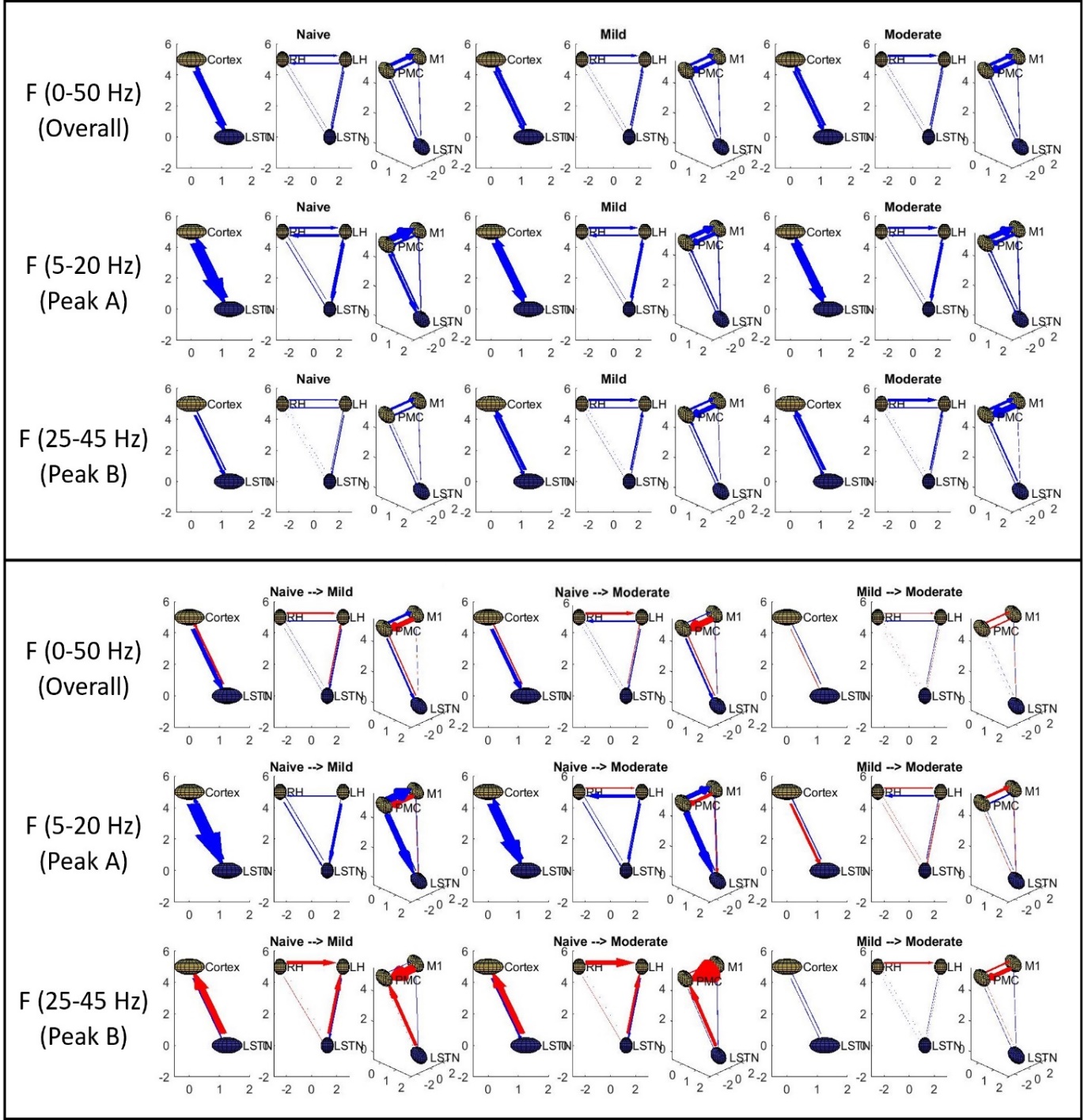


**(A)**

**(B)**

**Figure S5.** Topographic diagrams representing Granger causality analysis of the Combined Model. The topography of the model parallels that of **Figure 3** in the main publication and the three sub-networks are, from left to right, taken from a coronal, coronal, and left-coronal-sagittal viewpoint. The three underlying networks in the Combined Model are all shown. **A.** Relative GC interaction strength across the three experimental states. Arrow thickness represents the strength of directed connectivity. **B.** Relative change in interaction strength during state transition. Red represents an increase in G-causality and blue represents a decrease in G-causality.


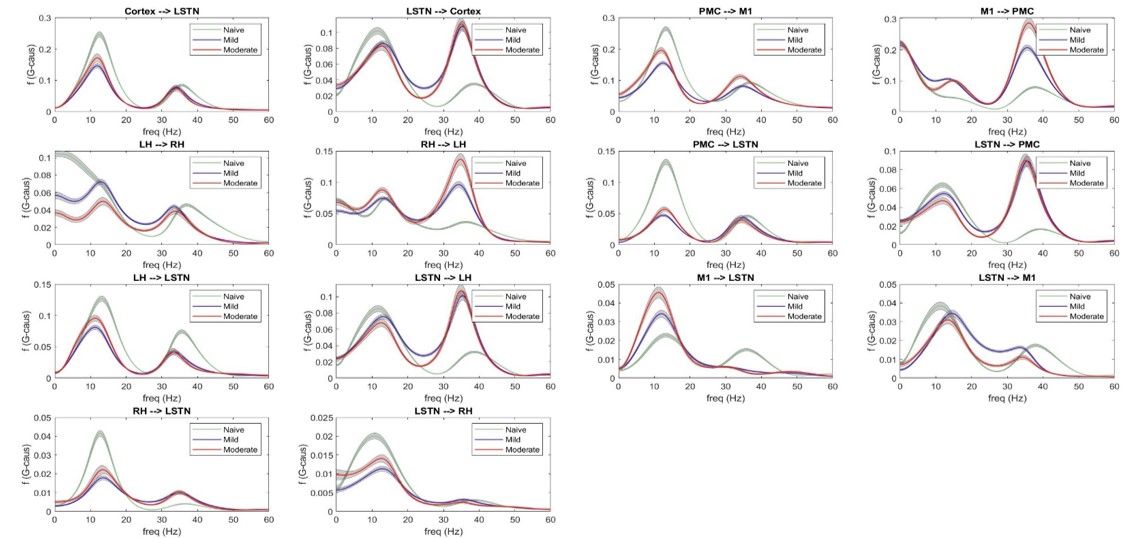

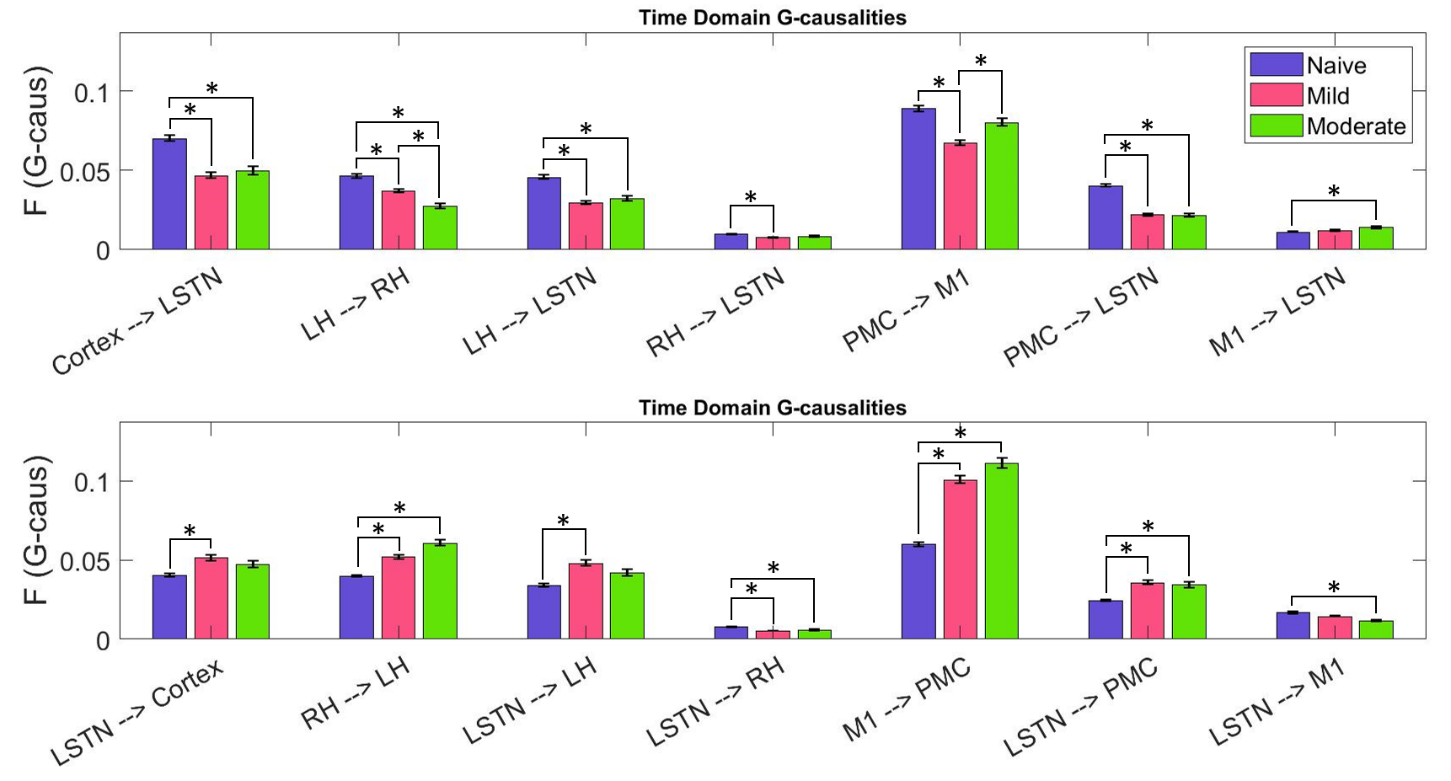
**Figure S6.** Frequency Domain Granger causality plots representing the Combined Model. This model exhibits the same frequency behavior as the Pairwise Model. However, the peaks in the Peak A and Peak B ranges are slightly more pronounced, and consistent in shape than for the Pairwise Model, as the interactions in the Combined Model are essentially those of the Pairwise Model integrated over multiple nodes. Shaded bars are an estimate of the 95% confidence interval, calculated as 1.96 times the standard error of the mean.

**Figure S7.** Time Domain G-causalities for the Combined Model. Overall G-causalities between 0-50 Hz. Note that these causalities are referenced to a common y-axis limit and so the magnitudes across interactions are relative to each other. Error bars represent standard error of the mean, and asterisks represent statistically significant differences between states at a significant level of α = 0.05.


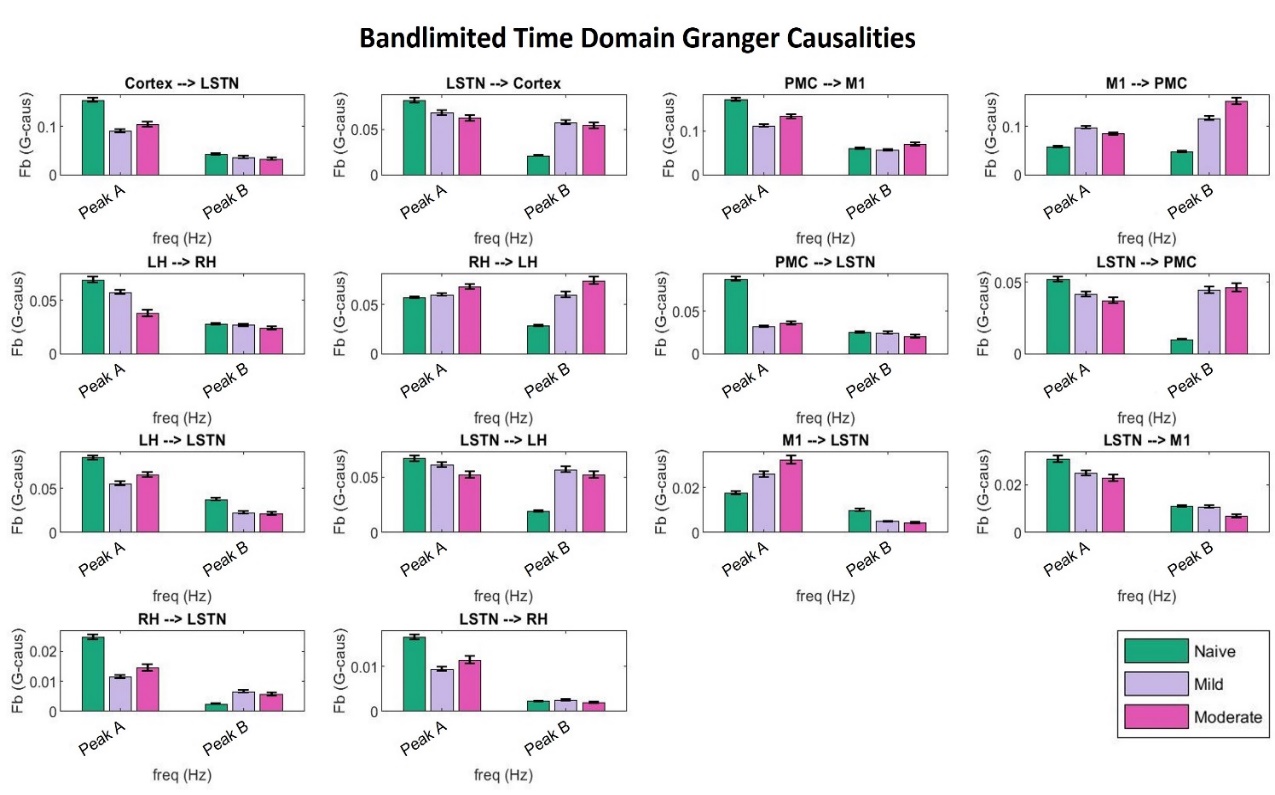
**Figure S8.** Bandlimited G-causalities for the Combined Model. The Peak A (5-20 Hz) G-causalities mainly decreased after the induction of Parkinsonism, and the Peak B (25-40 Hz) G-causalities mainly increased after disease onset. Error bars represent standard error of the mean. Analysis of statistical differences between experimental states was not conducted for these metrics for the Combined Model, and so there are no asterisks.

The shifts in G-causality which occurred in the Combined Model agree with those of the Pairwise Model, and faithfully reflect the same underlying process. However, the summative nature of this model allows for it to provide a different angle from which the data can be seen, which is more macroscopic and general.

Like in the Pairwise Model, significant changes were seen in both cortico-subthalamic and cortico-cortical interactions. The most general view of the shifts in cortico-subthalamic connectivity can be seen in the Cortex 🡨🡪 L-STN pair. The changes seen here are similar to those observed above, with the cortex-leading interaction (Cortex 🡪 L-STN) decreasing in the Peak A range and the STN leading interaction (L-STN 🡪 Cortex) increasing Peak B range after the onset of mild disease. During the Mild PD 🡪 Moderate PD transition, a slight increase in the Cortex 🡪 STN interaction is seen in the Peak A band, although this does not nearly offset the decrease which was accrued over the Naïve 🡪 Mild PD transition. The changes in connectivity between this pair are more modest than for the L-STN 🡨🡪 L-PMC pairwise causalities, however, because they are integrated across all four cortical nodes, most of which saw little change. The changes in G-causality between the LH 🡨🡪 L-STN and RH 🡨🡪 L-STN pairs show that, as expected, the observed changes occur exclusively in the left hemisphere. The PMC 🡨🡪 L-STN and M1 🡨🡪 L-STN interactions show, also in line with the results for the Pairwise Model, that the observed reciprocal effects occurred within the premotor cortex. These two results are in line with the findings of the Pairwise Model, which showed the left premotor cortex to be the node which was mainly connected to the L-STN.

The cortico-cortical interactions in the Combined Model are useful for making sense of those seen for the Pairwise Model in a simpler fashion. This model effectively reduces the twelve unique cortico-cortical pairwise interactions into just four summative interactions occurring between two pairs of nodes. These pairs are LH 🡨🡪 RH and PMC 🡨🡪 M1. The shifts in Granger causality allow for several general trends to emerge from the model. First, it shows that there is an overall increase in the magnitude of causality of the right hemisphere on the left hemisphere (RH 🡪 LH) and a decrease in the opposite direction (LH 🡪 RH). Consistent with findings reported above, the increases occur largely in the Peak B band and the decreases in the Peak A band. These changes also intensify significantly during both chronological transitions in disease state. Regarding the other pair of nodes, an increase was seen for the interaction of the primary motor cortex on premotor cortex (M1 🡪 PMC) in the Peak B band and a decrease was seen in the opposite direction (PMC 🡪 M1) in the Peak A band. The increase in M1 🡪 PMC causality occurred during both transitions, and the decrease in PMC 🡪 M1 causality occurred entirely within the Naïve 🡪 Mild PD transition, and then was partially reversed during the Mild PD 🡪 Moderate PD transition. These results are also consistent with those reported for the pairwise interactions.

The results for the Combined Model help to emphasize another general shift in the network which can actually be more properly seen in the Pairwise Model: after the induction of Parkinsonism, causalities which are directed towards the left premotor cortex (L-PMC) generally increase in the Peak B band, whereas causalities directed towards the right primary motor cortex (R-M1) generally decrease in the Peak A band.

Again, it is important that the changes in this model are understood in the context of their cumulative effect on the absolute magnitudes of the causalities in each of the experimental states. The changes in the Cortex 🡨🡪 L-STN causalities lead to a modest decrease in the Cortex 🡪 L-STN causality and an overall shift, or transfer, of causal magnitude from the Peak A to Peak B range. The corresponding increase in L-STN 🡪 Cortex causality was only salient in the Peak B band, which likely has to do with the dilution of causal effect caused by integrating over all four cortical channels. The increase in RH 🡪 LH causality and reciprocal decrease in LH 🡪 RH causality were large enough such that the LH 🡪 RH causality went from being slightly greater than its opposing causality in the Naïve state to significantly less than it in the Moderate PD state. The other cortical pair showed a similar reversal in dominance, with the M1 🡪 PMC causality becoming larger than the PMC 🡪 M1 causality after the induction of the disease. This primary motor cortex driven dominance is of similar strength across both disease states, however, the connectivity in both directions is strengthened by a similar amount in the moderate state relative to the mild state.

Regarding statistical inference for the Combined Model, all causal interactions tested as significantly different from zero, across all experimental states and across all frequency bands of interest. This likely has to do with the fact that for each of these interactions, at least one underlying pair of nodes that saw a significant causal effect in the Pairwise Model is included, and so the integration of that underlying causal effect with the other nodes in the macroscopic, or combined interaction caused there to be a significant causal effect.

## Combined Model Granger Causality – Transition Statistics

**Table S1** displays the statistical results for the transition between experimental states in the Combined Model. For more details on this analysis, see **Section 2.9** of the main paper. Note that this analysis was only conducted for the overall GC metric (0-50 Hz), and not for the bandlimited metrics (Peak A and Peak B).

**Table S1:** Adjusted p-values, Combined Model Granger Causality Analysis (bold, colored is significant at α = 0.05)

| Red = Increase  Blue = Decrease | Overall (0-50 Hz) | | |
| --- | --- | --- | --- |
|  | Naïve 🡪 Mild | Naïve 🡪 Moderate | Mild 🡪 Moderate |
| Cortex 🡪 L-STN | **.0020** | **.0168** | .9111 |
| L-STN 🡪 Cortex | **.0003** | .1700 | .1700 |
| LH 🡪 RH | **.0027** | **.0003** | **.0095** |
| RH 🡪 LH | **.0003** | **.0003** | .1059 |
| LH 🡪 L-STN | **.0003** | **.0027** | .8301 |
| L-STN 🡪 LH | **.0003** | .1001 | .1932 |
| RH 🡪 L-STN | **.0020** | .0774 | .5534 |
| L-STN 🡪 RH | **.0003** | **.0035** | .4806 |
| PMC 🡪 M1 | **.0003** | .0499 | **.0320** |
| M1 🡪 PMC | **.0003** | **.0003** | .3570 |
| PMC 🡪 L-STN | **.0003** | **.0003** | .6813 |
| L-STN 🡪 PMC | **.0003** | **.0046** | .5157 |
| M1 🡪 L-STN | .5063 | **.0316** | .1001 |
| L-STN 🡪 M1 | .1322 | **.0191** | .1926 |

# References

1. Barnett L, Barrett AB, Seth AK. Granger causality and transfer entropy are equivalent for Gaussian variables. Phys Rev Lett. 2009 Dec 4;103(23):238701. doi: 10.1103/PhysRevLett.103.238701. Epub 2009 Dec 4. PMID: 20366183.
2. Chen X, Zhang Y, Cheng S, Xie P. Transfer Spectral Entropy and Application to Functional Corticomuscular Coupling. IEEE Trans Neural Syst Rehabil Eng. 2019 May;27(5):1092-1102. doi: 10.1109/TNSRE.2019.2907148. Epub 2019 Mar 25. PMID: 30908233.
3. Li, F., Yi, C., Song, L. et al. Brain Network Reconfiguration During Motor Imagery Revealed by a Large-Scale Network Analysis of Scalp EEG. Brain Topogr 2019, 32:304–314.
4. Lindner M, Vicente R, Priesemann V, Wibral M. TRENTOOL: a Matlab open source toolbox to analyse information flow in time series data with transfer entropy. BMC Neurosci. 2011 Nov 18;12:119. doi: 10.1186/1471-2202-12-119. PMID: 22098775; PMCID: PMC3287134.
5. Marinazzo D, Pellicoro M, Stramaglia S. Kernel method for nonlinear granger causality. Phys Rev Lett. 2008 Apr 11;100(14):144103. doi: 10.1103/PhysRevLett.100.144103. Epub 2008 Apr 11. PMID: 18518037.
6. Pinzuti E, Wollstadt P, Gutknecht A, Tüscher O, Wibral M. Measuring spectrally-resolved information transfer. PLoS Comput Biol. 2020 Dec 28;16(12):e1008526. doi: 10.1371/journal.pcbi.1008526. PMID: 33370259; PMCID: PMC7793276.
7. Schreiber T. Measuring information transfer. Phys Rev Lett. 2000 Jul 10;85(2):461-4. doi: 10.1103/PhysRevLett.85.461. PMID: 10991308.
